# Syntheses of Precursors and Reference Compounds of the Melanin-Concentrating Hormone Receptor 1 (MCHR1) Tracers [^11^C]SNAP-7941 and [^18^F]FE@SNAP for Positron Emission Tomography

**DOI:** 10.3390/molecules181012119

**Published:** 2013-09-30

**Authors:** Eva Schirmer, Karem Shanab, Barbara Datterl, Catharina Neudorfer, Markus Mitterhauser, Wolfgang Wadsak, Cécile Philippe, Helmut Spreitzer

**Affiliations:** 1 University of Vienna, Department of Drug and Natural Product Synthesis, Althanstraße 14, 1090 Vienna, Austria; 2 Medical University of Vienna, Department of Biomedical Imaging and Image-guided Therapy, Division of Nuclear Medicine, Währinger Gürtel 18-20, 1090 Vienna, Austria; 3 Hospital Pharmacy of the General Hospital of Vienna, Währinger Gürtel 18-20, Vienna 1090, Austria; 4 University of Vienna, Department of Pharmaceutical Technology and Biopharmaceutics, Althanstraße 14, 1090 Vienna, Austria; 5 University of Vienna, Department of Inorganic Chemistry, Währinger Str. 42, 1090 Vienna, Austria

**Keywords:** MCH receptor 1 (MCHR1), SNAP-7941, *rac*SNAP-7941, positron emission tomography, adiposity

## Abstract

The MCH receptor has been revealed as a target of great interest in positron emission tomography imaging. The receptor′s eponymous substrate melanin-concentrating hormone (MCH) is a cyclic peptide hormone, which is located predominantly in the hypothalamus with a major influence on energy and weight regulation as well as water balance and memory. Therefore, it is thought to play an important role in the pathophysiology of adiposity, which is nowadays a big issue worldwide. Based on the selective and high-affinity MCH receptor 1 antagonist SNAP-7941, a series of novel SNAP derivatives has been developed to provide different precursors and reference compounds for the radiosyntheses of the novel PET radiotracers [^11^C]SNAP-7941 and [^18^F]FE@SNAP. Positron emission tomography promotes a better understanding of physiologic parameters on a molecular level, thus giving a deeper insight into MCHR1 related processes as adiposity.

## 1. Introduction

Reports have suggested that the melanin-concentrating hormone (MCH), a cyclic peptide located predominantly in the hypothalamus, plays a significant role in regulation of food intake and stress in rodents. This hormone, which consists of 19 amino acids, regulates physiological functions such as water balance, energy metabolism, general arousal, attention state and is also assumed to be involved in memory and psychiatric disorders, although its role therein still remains largely unknown [1,2,3,4,5,6].

MCH-producing neurons innervate vast parts of the brain [1,2,3,4,5,6], but the lateral hypothalamus where most of these neurons have been localized, is considered as the regulatory center for food intake, body temperature, blood pressure, rhythm of sleep, and the reward center which is closely connected with emotions [5]. Animal experiments with MCH overexpressing mice proved the correlation between adiposity and MCH expression: compared with genetic unmodified mice, MCH-OE mice were hyperphagic, hyperleptinaemic, and had higher blood concentrations of glucose. Additionally, these animals were significantly hyperinsulinaemic and showed insulin resistance after insulin injection [3,7,8].

**Figure 1:**
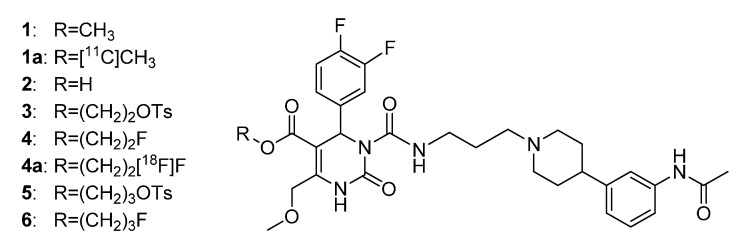
Structure of SNAP-7941 and derivatives **1a**–**6**.

As described by Kokkotou *et al* [8], the adipose-derived hormone leptin determines the regulation of the expression of MCH, other hypothalamic hormones, and the expression of the MCH receptor (MCHR). By gaining deeper insight in the function of the MCHR1 through positron emission tomography (PET), useful information about adiposity can be obtained for future research [3,9]. PET is an important tool both in medical diagnostics and clinical research of molecular processes due to its non-invasive nature as an imaging technique. Based on the already established selective, high-affinity MCHR1 antagonist SNAP-7941 (**1**), which has anorectic, antidepressant, and anxiolytic effects [10,11,12,13,14], the present study aimed at the synthesis and evaluation of precursors and reference standards of the novel MCH receptor 1 PET tracers [^11^C]SNAP-7941 (**1a**) and [^18^F]FE@SNAP (**4a**) [15,16] (Figure 1).

In particular, this paper focuses on the synthesis of the novel MCHR1 PET tracers’ **1a** and **4a**, non-radioactive reference compound FE@SNAP **4** as well as the precursors SNAP-acid **2** and TOE@SNAP **3**, which represents the preliminary non-radioactive work paving the way for the subsequent radiosyntheses [15,16].

Compounds **2**, **3**, and **5** can either serve as precursors for radioactive labeling or regarding **3** for non-radioactive fluorination. The reference compounds **1**, **4**, and **6** serve as standards for the quality control of the radiosyntheses. Regarding the tracer[^11^C]SNAP-7941 (**1a**), *rac*SNAP-7941 **1** [10,11,12,13,14] can be used as a reference compound. *In-vivo* studies, biodistribution, and micro PET investigations of the radiotracers[^11^C]SNAP-7941 **1a** and [^18^F]FE@SNAP **4a** are going to be future challenges directly based on this work.

## 2. Results and Discussion

**Scheme 1:**
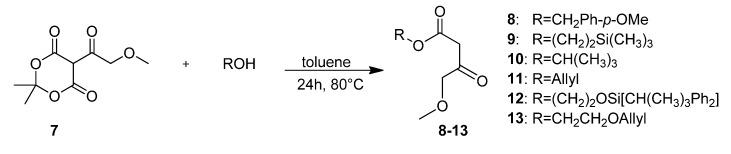
Syntheses of β-ketoesters **8**–**13**.

All SNAP derivatives and intermediates were produced as racemates, deviating from Borowsky *et al* [1]. The complete reaction sequence is depicted in Scheme 1–Scheme 14. Instead of using methoxymethyl acetoacetate as a starting material for the subsequent Biginelli cyclization, a series of different beta-ketoesters **8**–**13** carrying different protecting groups for easier cleavage was synthesized (Scheme 1).

**Figure 2:**
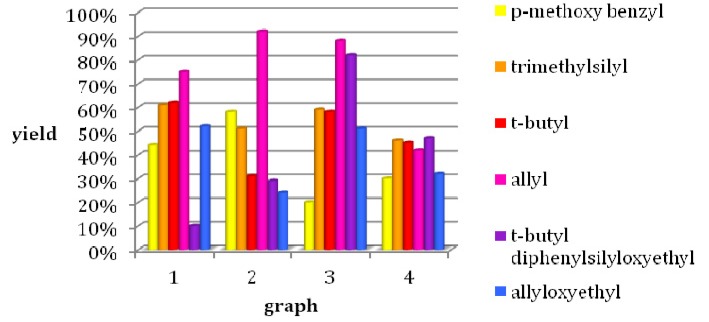
Yields for different steps in the synthesis of the six SNAP derivatives. Graph 1: synthesis of β-ketoesters **8**–**13**; graph 2: Biginelli cyclization of **15**–**20**; graph 3: synthesis of **22**–**27** in a one-pot two-step reaction; graph 4: synthesis of the SNAP derivatives **29**–**34**.

Therefore, the first step of the reaction pathway was the preparation of 5-(methoxyacetyl)-2,2-dimethyl-1,3-dioxane-4,6-dione) (**7**) from Meldrum′s acid, which was then reacted with altogether six different alcohols in toluene at 80 °C overnight to give β-ketoesters **8**–**13**. Depending on the alcohol, six different protecting groups were attached as esters: *p*-methoxybenzyl, 2-(trimethylsilyl)ethyl, *t-*butyl, 2-(allyloxy)ethyl and 2-(*tert*-butyldiphenyl-silyloxy)ethyl. The comparison of yields is given in Graph 1 of Figure 2.

**Scheme 2:**
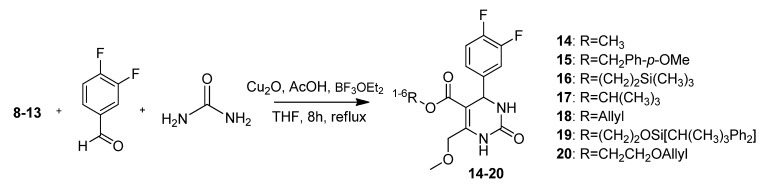
Biginelli cyclizations.

As shown in Scheme 2, Scheme 4, Scheme 5 and Scheme 6, the synthesis of *rac*SNAP-7941 **1** was accomplished according to the literature without any modifications to the reaction conditions [17]. Derivatives **29**–**34** have been substituted with different protecting groups instead of the methyl ester moiety. The Biginelli cyclization reaction was conducted based on an alternative method of Murali Dhar *et al.* [18]. SNAP derivatives **29**–**32** were used for the synthesis of the precursor SNAP-acid **2**, compounds **33** and **34** served as starting material for the hydroxyethyl derivative **35**, as depicted in Scheme 10. 

The syntheses leading to **2** and the allyl protected derivatives **11**, **18**, **25**, and **32** were performed as already described by Philippe *et al.* [15], as were those of compounds **3** and **4** [16]. The syntheses of the already known compounds **1**, **14**, **21** and **28** were carried out according to Schönberger [17]. For completeness of this paper, they are depicted in Scheme 2, Scheme 4, Scheme 5 and Scheme 6 as well.

In the next step, a Biginelli reaction was performed using urea, the respective beta ketoesters **8**–**13** or methoxymethyl acetoacetate, and difluorobenzaldehyde as starting materials, followed by addition of copper oxide, acetic acid, and boron trifluoride diethyl etherate in THF. The mixtures were refluxed for 8 hours to give the seven different pyrimidinones **14**–**20** (Scheme 2).

Figure 2/Graph 2 shows a comparison of the different yields of pyrimidinones **15**–**20** related to the protecting groups. Cyclization using the *t*-butylester **10**, for example, gave only 30% of the corresponding pyrimidinone **17**, whereas the best yields were accomplished using the allyl protected ester **11**. Allyl pyrimidinone **18** was obtained in an excellent 90% yield.

**Scheme 3:**
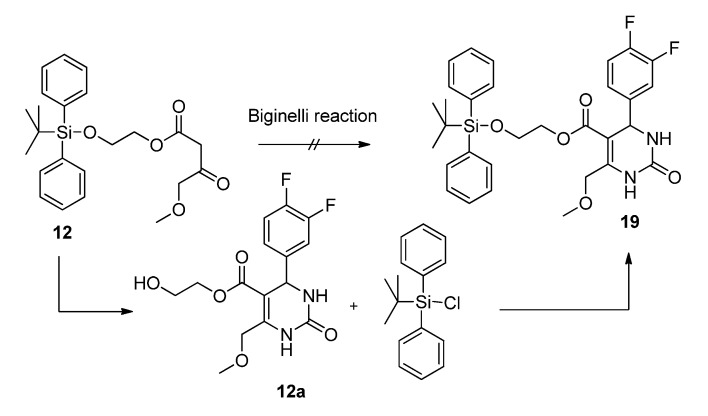
Biginelli cyclization with *t*-butyltrimethylsilyloxyethyl protected ester **12**.

Unfortunately, while reacting the *t-*butyltrimethysilyloxyethyl protected ester **12** the protecting group was cleaved during the cyclization step, affording hydroxyethyl pyrimidinone **12a** as shown in Scheme 3. Hence, the protecting group had to be reattached in an additional step.

**Scheme 4:**
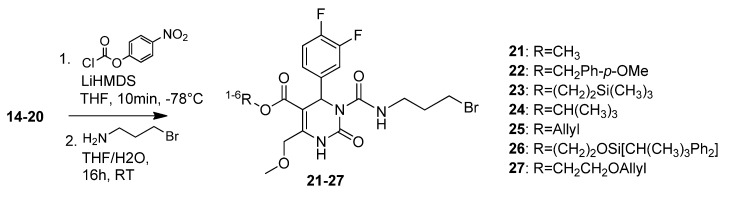
Attachment of the 3-bromopropylcarbamoyl side chain to pyrimidinones **14**–**20**.

A 3-bromopropylcarbamoyl side chain was attached to the pyrimidinones **14**–**20** in a one-pot two-step reaction, yielding compounds **21**–**27** (Scheme 4). Again, the allyl protected compound **25** was obtained in excellent yields of 85% as shown in Figure 2/Graph 3.

**Scheme 5:**
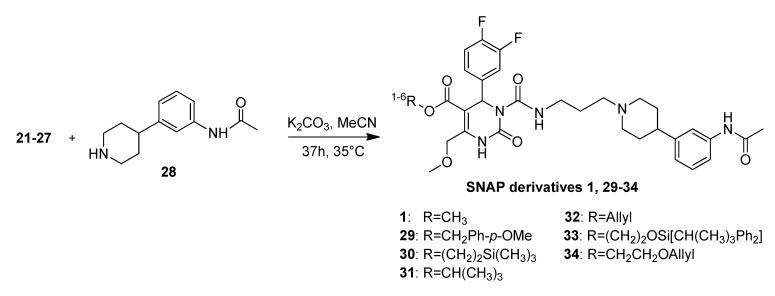
Syntheses of the SNAP derivatives **1**, **29**–**34**.

The side chain **28** was attached onto compounds **21**–**27** by addition of potassium carbonate, giving *rac*SNAP-7941 **1** or SNAP derivatives **29**–**34** (Scheme 5), respectively, exhibiting a similar pattern of yields as in the previous reactions in correlation to the corresponding protection group (Figure 2/Graph 4).

**Scheme 6:**
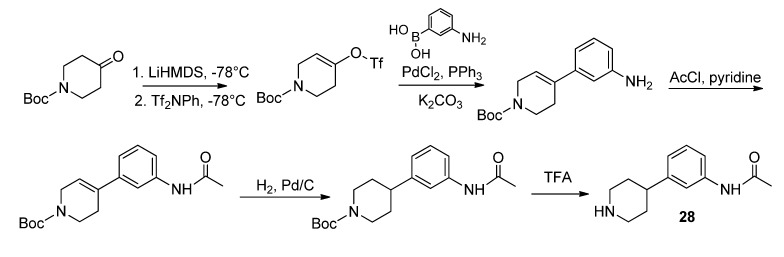
Synthesis of N-(piperidinyl)acetamide compound **28**.

The *N*-(piperidinylphenyl)acetamide side chain **28** was obtained according to Schönberger [17] via Suzuki coupling, hydrogenation and acetylation followed by cleavage of the *t*-butyloxycarbonyl protecting group as shown in Scheme 6.

**Scheme 7:**
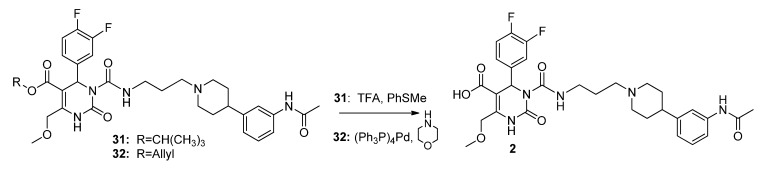
Cleavage of esters **31** and **32** to furnish SNAP-acid **2**.

Compounds **29**–**32** were subjected to cleavage reactions in order to obtain SNAP-acid **2**. Unfortunately, only the *t*-butyl protected compound **31** and the allyl protected compound **32** could be converted into the free carboxylic acid **2** (Scheme 7).

**Scheme 8:**
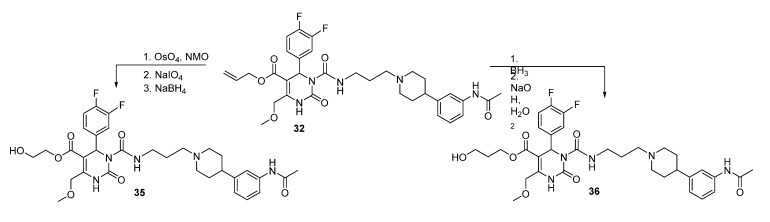
Synthesis of hydroxylethyl and hydroxypropyl esters **35** and **36**.

In total, regarding the superior yields of **32** as shown in Figure 2, the synthesis of allyl ester **32** was established as the most effective route of preparing the PET precursor SNAP-acid **2**. Additionally, allyl ester **32** served as starting material for the hydroxyethyl ester HE@SNAP **35** as well as for the hydroxypropyl ester HP@SNAP **36**, which were subjected to tosylation for subsequent fluorination (Scheme 8). The tosylated compounds **3** and **5** were prepared as two alternative precursors of the desired target compounds **4** and **6**, in order to compare the feasibility of fluorination of the tosyl ethyl derivative **3** to the tosyl propyl derivative **5**.

**Scheme 9:**
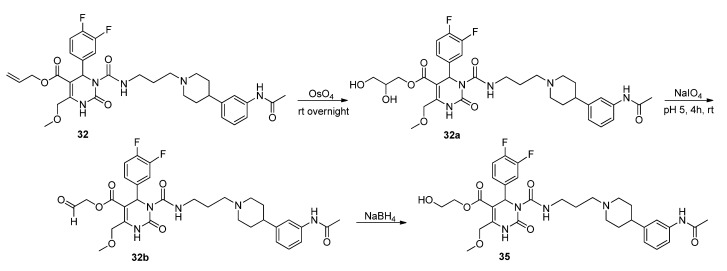
Synthesis of hydroxyethyl ester **35** via glycol cleavage.

The synthesis of **35** required three reaction steps, starting with the oxidation of the allyl protecting group using osmium tetroxide performed by adapting and combining different methods [19,20,21]. This yielded 2,3-dihydroxypropyl ester **32a**, as depicted in Scheme 9.

**Scheme 10:**
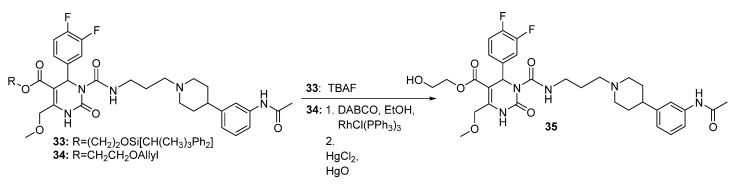
Synthesis of hydroxyethyl ester **35** via SNAP derivatives **33** and **34**.

Then, a glycol cleavage of the 2,3-dihydroxypropyl group was performed with sodium periodate adapting methods of Botti *et al*. [22] and Adam *et al*. [23] to yield aldehyde **32b**, which was subjected to reduction under standard conditions with sodium borohydride [24] to give 2-hydroxylethyl ester **35**.

The hydroxypropyl analogue **36** was synthesized in a one-pot two-step reaction as shown above in Scheme 9 adapting the methods of Heidecke/Lindhorst and Park *et al*. [25,26]. The cleavage to hydroxypropyl ester **36** was accomplished in an anti-Markovnikov reaction using a borane-tetrahydrofuran complex and hydrogen peroxide. Although the unconsumed starting material could be partially recovered by column chromatography, the reaction afforded only a moderate 26% yield. A second and third approach to HE@SNAP **35** was made accessible by the cleavage of the protecting groups of SNAP derivatives **33** and **34**, respectively (Scheme 10).

**Scheme 11:**
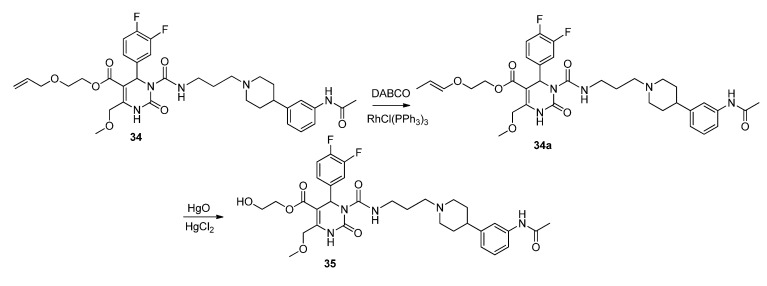
Reaction sequence to hydroxyethyl ester **35**.

The protecting group of compound **33** was cleaved in a standard procedure [27] using tetrabutyl-ammonium fluoride, while the allyloxyethyl ester **34** had to be isomerized first with a Wilkinson catalyst as depicted in Scheme 11. Isomerization of the allyl group was conducted in the presence of diazabicyclooctane and the catalyst, adapting a method of Smith *et al*. [28]. Mercury-induced cleavage of the newly formed vinyl ether [29] give satisfying yields, regarding the feasibility of recycling the starting material.

**Scheme 12:**
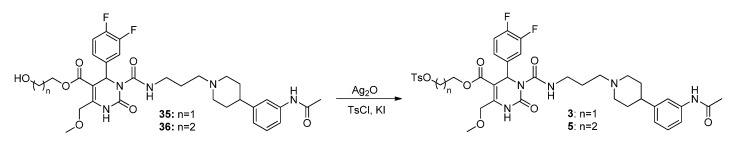
Preparation of tosylated SNAP derivatives **3** and **5**.

Compounds **35** and **36** were used for tosylation yielding 2-(tosyloxy)ethyl ester TOE@SNAP **3** and 3-(tosyloxy)propyl ester TOP@SNAP **5**, respectively. Since common tosylation methods [30,31,32] were not applicable, tosylation was achieved using silver oxide and tosyl chloride in the presence of potassium iodide adapting a method of Bouzide *et al*. [33]. Comparing the yields, TOE@SNAP **3** was obtained with 63%, whereas the tosylpropyl derivative **5** gave a poorer 31% yield (Scheme 12).

**Scheme 13:**
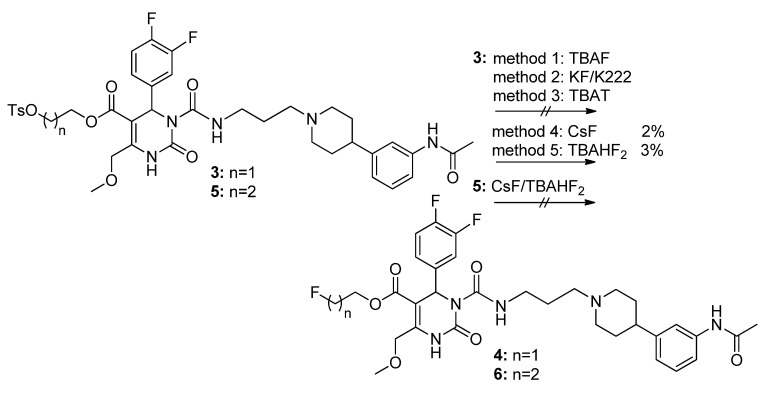
Conversion of tosylated SNAP precursors **3** and **5**.

The tosylated derivatives **3** and **5** were intended to be used for the following fluorination to afford the final compounds **4** and **6** (Scheme 13).

Unfortunately, different fluorination methods such as reactions with tetrabutylammonium fluoride [30], crown ether Kryptofix® K2.2.2 and potassium fluoride [34], or tetrabutylammonium-(triphenylsilyl) difluorosilicate [35] were unsuccessful. Minor yields of **4** (2%–3%) could be obtained by fluorination with cesium fluoride [36] and tetrabutylammoniumhydrogen difluoride [37]. Conversion of compound **5** to fluoropropyl ester **6** under similar reaction conditions was confirmed by high resolution mass spectrometry (HRMS) analysis but purification and isolation could not be accomplished due to the probable instability of this product. Attempting to react the hydroxyethyl derivative **35** with diaminosulfur trifluoride by adapting a method of Shanab [30] did not provide the fluoroethyl ester **4** either.

**Scheme 14:**
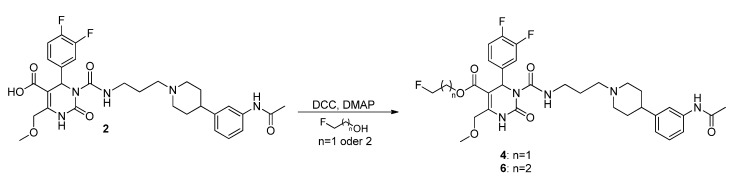
Conversion of SNAP-acid **2** to fluorinated derivatives **4** and **6**.

Hence, another approach to **4** and **6** had to be established leading to the Steglich esterification [16,38,39] of SNAP-acid **2** (Scheme 14) which is therefore employed as a precursor for the reference compound FE@SNAP **4**, for the PET tracer[^11^C]SNAP-7941 **1a** via 11C-methylation [15], and for the second tracer [^18^F]FE@SNAP **4a** [16].

Acid **2** was reacted with dicyclohexylcarbodiimide, 4-dimethylaminopyridine, and fluoroethanol or fluoropropanol, respectively, giving the fluoroethylated reference compound **4** in trace yields of 4% but again did not afford **6** in acceptable quantity, although the conversion was confirmed via mass spectrometry. Fluoropropyl ester **6** could not be isolated by different chromatographic purification methods. Therefore, the synthesis of the propylated compounds **36**, **5**, and **6** was not further pursued due to the better yields and superior purification properties of the ethylated compounds **35**, **3**, and **4**.

## 3. Experimental

### 3.1. General

All commercial chemicals and solvents used in the synthetic steps were purchased from Aldrich (Vienna, Austria) or Fisher Scientific (Vienna, Austria) and used as received. Reactions were monitored by thin layer chromatography (TLC) using appropriate developing solvents and pre-coated silica gel plates (UV 254 nm) purchased from Merck and Co. (Vienna, Austria). ^1^H-NMR and ^13^C-NMR spectra were recorded on a Bruker Avance DPX-200 spectrometer,a Varian UnityPlus 500 spectrometer or a Bruker Avance 500 spectrometer. Chemical shifts are reported in δ (ppm) relative to tetramethylsilane (TMS) as internal standard and multiplicities are given as singlet (s), doublet (d), quartet (q), multiplet (m) and broad singlet (br s). IR-spectra were recorded on a Perkin Elmer FT-IR Spectrum 1000 spectrophotometer. High resolution mass spectral data were obtained on a Finnigan MAT 8230 or on a Finnigan MAT 900 S. Elemental analyses were performed at the Mass Spectrometry Centre of the Faculty of Chemistry (University of Vienna).

### 3.2. Syntheses

Synthesis of compounds **1**, **14**, **21** and **28** was conducted according to Schönberger [17]. Synthesis of compounds **2**, **7**, **11**, **18**, **25** and **32** was conducted according to Philippe *et al.* [15]. Synthesis of compounds **3** and **4** was conducted according to Philippe *et al.* [16].

*3-(Tosyloxy)propyl-3-(3-(4-(3-acetamidophenyl)piperidin-1-yl)propyl-carbamoyl)-4-(3,4-difluoro-phenyl)-6-(methoxymethyl)-2-oxo-1,2,3,4-tetrahydropyrimidine-5-carboxylate* (**5**). To a stirred solution of alcohol **36** (116 mg, 0.18 mmol) in CH_2_Cl_2_ (1.0 mL), freshly produced Ag_2_O (83 mg, 0.36 mmol), tosyl chloride (69 mg, 0.36 mmol) and KI (60 mg, 0.36 mmol) were added. The mixture was stirred at 40 °C until completion of the reaction (TLC-monitoring). Thereafter, the reaction mixture was filtered and the solvent evaporated *in vacuo*. The residue was purified via column chromatography (silica gel, eluent: CH_2_Cl_2_/MeOH 9:1) to give 23 mg (26.1%) of product **5**. ^1^H-NMR (200 MHz, CDCl_3_): δ (ppm) 1.87–1.97 (m, 8H, 9b-CH_2_, 19-CH2, 22,22′-(CH_2_)_2_), 2.05–2.08 and 3.08–3.15 (m, 4H, 21,21′-(CH_2_)_2_), 2.15 (s, 3H, 32-CH_3_), 2.42–2.51 (m, 6H, Tos-CH_3_, 20-CH_2_, 23-CH), 3.37–3.40 (m, 2H, 18-CH_2_), 3.45 (s, 3H, 7-OCH_3_), 3.94–4.04 (m, 2H, 9c-OCH_2_), 4.10–4.18 (m, 2H, 9a-OCH_2_), 4.65 (s, 2H, 6-OCH_2_), 6.58 (s, 1H, 3-CH), 6.92 (d, 1H, *J* = 7.2 Hz, 29-CH), 7.04–7.26 (m, 5H, 11-CH, 14-CH, 15-CH, 27-CH, 28-CH), 7.30-7.34 (m, 2H, 3′,3′′-(CH)_2_), 7.44 (s, 1H, 30-NH), 7.72–7.76 (m, 2H, 2′,2′′-(CH)_2_), 7.99 (s, 1H, 1-NH), 8.96 (t, 1H, J = 5.2 Hz, 17-NH); ^13^C-NMR (50 MHz, CDCl_3_): δ (ppm) 21.6 (Tos-CH_3_), 24.5 (32-CH_3_), 26.0 (19-CH_2_), 29.6 (9b-CH_2_), 32.4 (22,22′-(CH_2_)_2_), 39.4 (18-CH_2_), 42.2 (23-CH), 52.9 (3-CH), 54.1 (21,21′-(CH_2_)_2_), 56.4 (20-CH_2_), 59.1 (7-OCH_3_), 60.4 (9c-CH_2_OH), 66.6 (9a-OCH_2_), 68.0 (6-OCH_2_), 101.2 (4-C), 116.0/116.3 (11-CH), 117.2/117.5 (14-CH), 117.7 (27-CH), 118.1 (25-CH), 122.7 (29-CH), 122.9/123.0/123.1/123.2 (15-CH), 127.8 (2′,2′′-(CH)_2_), 128.9/129.0 (28-CH), 129.9 (3′,3′′-(CH)_2_), 132.7 (1′-C), 137.5 (10-C), 138.3 (26-C), 144.9 (4′-C), 146.6 (5-C), 146.8 (24-C), 152.1 (2-CO), 153.2 (16-CO), 163.8 (8-COO), 168.6 (31-CON); MS: *m/z* (%) 812 (1), 371 (46), 286 (56), 231 (43), 71 (29), 70 (100), 65 (28), 57 (38), 56 (55); HRMS: Calcd. for C_40_H_48_F_2_N_5_O_9_S [M + H]^+^: 812.3141. Found: 812.3148.

*4-Methoxybenzyl 4-methoxy-3-oxobutanotate* (**8**). 5-(2-Methoxyacetyl)-2,2-dimethyl-1,3-dioxane-4,6-dione (7, 724 mg, 3.35 mmol) and (4-methoxyphenyl)methanol (925 mg, 6.69 mmol) in toluene (10.0 mL) were heated to 80 °C for 24 h. After cooling to room temperature the solvent was removed *in vacuo* and the residue was partly purified by column chromatography (silica gel, eluent: petroleum ether/EtOAc 3:1) to give 372 mg (44.0%) of **8** as a brown oil. The crude product was reacted without further purification in the next step.

*2-(Trimethylsilyl)ethyl 4-methoxy-3-oxobutanoate* (**9**). A mixture of 5-(2-methoxyacetyl)-2,2-dimethyl-1,3-dioxane-4,6-dione (**7**, 3.60 g, 16.65 mmol) and 2-(trimethylsilyl)ethanol (4.8 mL, 3.94 g, 33.32 mmol) in toluene (50.0 mL) was heated to 80 °C for 24 h. After cooling to room temperature the solvent was removed *in vacuo* and the residue was purified by column chromatography (silica gel, eluent: petroleum ether/EtOAc 9:1) to give 2.35 g (61.0%) of **9** as a light brown oil. ^1^H-NMR (200 MHz, CDCl_3_): δ (ppm) −0.01 (s, 9H, Si(CH_3_)_3_), 0.91–1.00 (m, 2H, SiCH_2_), 3.36 (s, 3H, OCH_3_), 3.43 (s, 2H, 2-CH_2_), 4.03 (s, 2H, 4-OCH_2_), 4.12–4.21 (m, 2H, OCH_2_); ^13^C-NMR (50 MHz, CDCl_3_): δ (ppm) −1.7 (Si(CH_3_)_3_), 17.1 (SiCH_2_), 45.8 (2-CH_2_), 59.2 (OCH_3_), 63.6 (OCH_2_), 77.2 (4-OCH_2_), 167.0 (1-COO), 201.5 (3-CO); MS *m/z* (%): 232 (1), 147 (8), 117 (8), 75 (64), 74 (12), 73 (100), 72 (9), 59 (9), 45 (12); HRMS: Calcd. for C_8_H_16_O_4_Si [M – C_2_H_4_]: 204.0818. Found: 204.0813.

*t-Butyl 4-methoxy-3-oxobutanoate* (**10**). 5-(2-Methoxyacetyl)-2,2-dimethyl-1,3-dioxane-4,6-dione (**7**, 6.40 g, 19.60 mmol) and 2-methylpropan-2-ol (9.00 g, 121.42 mmol) were dissolved in toluene (90.0 mL) and the mixture was stirred for 24 h at 80 °C. After cooling to room temperature, the solvent and excess alcohol were removed *in vacuo* and the residue was purified by column chromatography (RP silica gel, eluent: ACN/H_2_O 9:1) and Kugelrohr distillation (b.p. ca 260 °C) to give compound **10** as a brown oil (3.44 g, 62.0%). ^1^H-NMR (200 MHz, CDCl_3_): δ (ppm) 1.40 (s, 9H, *t-*but-(CH_3_)_3_), 3.34 (s, 2H, 2-CH_2_), 3.36 (s, 3H, 5-OCH_3_), 4.02 (s, 2H, 4-CH_2_); ^13^C-NMR (50 MHz, CDCl_3_): δ (ppm) 27.8 (*t-*but-(CH_3_)_3_), 47.0 (2-CH_2_), 59.1 (OCH_3_), 77.1 (4-OCH_2_), 81.9 (*t-*but-C), 166.0 (1-COO), 201.8 (3-CO); MS: *m/z* (%) 132 (5), 115 (6), 87 (4), 69 (5), 59 (33), 58 (8), 57 (100), 56 (13), 45 (51), 43 (16), 42 (11), 41 (26); HRMS: Calcd. for C_5_H_8_O_4_ [M – C_4_H_8_]: 132.0423. Found: 132.0424.

*2-(t-Butyldiphenylsilyloxy)ethyl 4-methoxy-3-oxobutanoate* (**12**). First, 2-(*t-*butyldiphenylsilyloxy)-ethanol was freshly prepared. To a mixture of ethylene glycol (5.59 g, 90.00 mmol), imidazole (6.13 g, 90.00 mmol), and absolute CH_2_Cl_2_ (140 mL) *t-*butylchlorodiphenylsilane (23.03 mL, 24.74 g, 90.00 mmol) dissolved in absolute CH_2_Cl_2_ was added dropwise at 0 °C. After stirring for 24 h at room temperature, the reaction mixture was extracted with water to remove unreacted ethylene glycol. The solvent was removed under reduced pressure to give 21.81 g (81.1%) of 2-(*t-*butyldiphenyl-silyloxy)ethanol as a colorless oil which crystallized upon cooling to afford colorless crystals. The crude product (21.69 g, 72.19 mmol) was used for the next step without further purification and heated to 80 °C for 24 h with 5.23 g (24.21 mmol) of 5-(2-methoxyacetyl)-2,2-dimethyl-1,3-dioxane-4,6-dione (**7**) in toluene (73.0 mL). After cooling to room temperature the solvent was removed *in vacuo* and the residue was purified by column chromatography (reversed-phase silica gel, eluent: acetonitrile/H_2_O 4:1→1:0) to give 3.10 g (10.4%) of **12** as reddish brown oil. ^1^H-NMR (200 MHz, CDCl_3_): δ (ppm) 1.07 (s, 9H, *t-*but-(CH_3_)_3_), 3.40 (s, 3H, OCH_3_), 3.49 (s, 2H, 2-CH_2_), 3.85–3.90 (m, 2H, 9b-OCH_2_), 4.08 (s, 2H, 4-OCH_2_), 4.24–4.29 (m, 2H, 9a-OCH_2_), 7.37–7.45 (m, 6H, 2′-(CH)_2_, 4′-(CH)_2_, 6′-(CH)_2_), 7.66–7.70 (m, 4H, 3′-(CH)_2_, 5′-(CH)_2_); ^13^C-NMR (50 MHz, CDCl_3_): δ (ppm) 19.1 (*t-*but-C), 26.7 (*t-*but-(CH_3_)_3_), 45.6 (2-CH_2_), 59.3 (OCH_3_), 61.7 (9b-OCH_2_), 66.3 (9a-OCH_2_), 77.2 (4-OCH_2_), 127.7 (3′-(CH)_2_, 5′-(CH)_2_), 129.7 (4′-(CH)_2_), 133.2 (1′-(C)_2_), 135.5 (2′-(CH)_2_, 6′-(CH)_2_), 166.9 (1-COO), 201.3 (3-CO); MS: *m/z* (%) 383 (4), 349 (3), 243 (18), 199 (89), 185 (24), 184 (48), 165 (65), 154 (42), 139 (24), 111 (56), 94 (23), 69 (33), 45 (100); HRMS: *m/z* calcd. for C_23_H_30_O_5_SiNa [M + Na]^+^: 437.1760. Found: 437.1760.

*2-Hydroxyethyl 4-(3,4-difluorophenyl)-6-(methoxymethyl)-2-oxo-1,2,3,4-tetrahydropyrimidine-5-carboxylate* (**12a**). To a well-stirred solution of allyloxyethyl 4-methoxy-3-oxobutanoate (**12**, 3.00 g, 7.24 mmol), 3,4-difluorobenzaldehyde (1.20 g, 8.44 mmol) and urea (0.73 g, 12.15 mmol) in THF (7.0 mL), Cu_2_O (117 mg, 0.82 mmol), glacial acetic acid (47 µL) and boron trifluoride diethyl etherate (1.29 mL, 1.46 g, 10.32 mmol) were added. The resulting reaction mixture was heated under reflux for 8 h. After cooling to room temperature the mixture was poured onto a mixture of ice (12 g) and NaHCO_3_ (2 g). The resulting cloudy solution was filtered over Celite and washed with CH_2_Cl_2_ (10 mL). The biphasic solution was separated in a separatory funnel and the aqueous phase was washed with CH_2_Cl_2_ (3 × 7 mL). The combined organic layers were dried over Na_2_SO_4_, filtered and evaporated *in vacuo*. The crude product was purified by column chromatography (silica gel, eluent: CH_2_Cl_2_/MeOH 9:1) to give 0.90 g (36.3%) of **12a** as yellow oil. ^1^H-NMR (200 MHz, CDCl_3_): δ (ppm) 3.44 (s, 3H, 7-OCH_3_), 3.71 (m, 2H, 9b-CH_2_OH), 4.12–4.20 (m, 2H, 9a-OCH_2_), 4.63 (s, 2H, 6-OCH_2_), 5.37 (d, *J* = 2.4 Hz, 3-CH), 6.77 (s, 1H, 2a-CH), 7.02–7.18 (m, 3H, 11-CH, 14-CH, 15-CH), 7.75 (s, 1H, 1-NH); ^13^C-NMR (50 MHz, CDCl_3_): δ (ppm) 54.3 (3-CH), 59.1 (7-OCH_3_), 60.9 (9b-CH_2_OH), 65.9 (9a-OCH_2_), 68.5 (6-OCH_2_), 98.2 (4-C), 115.4/115.8 (11-CH), 117.3/117.7 (14-CH), 122.4/122.5 (15-CH), 140.5 (10-C), 148.2 (5-C), 152.3 (2-CO), 164.8 (8-COO); MS: *m/z* (%) 342 (22), 310 (14), 280 (33), 267 (74), 253 (27), 229 (100), 221 (24), 167 (50), 153 (38), 45 (40); HRMS: *m/z* calcd. for C_15_H_16_F_2_N_2_O_5_Na [M + Na]^+^: 365.0925. Found: 365.0932.

*Allyloxyethyl 4-methoxy-3-oxobutanoate* (**13**). A mixture of 5-(2-methoxyacetyl)-2,2-dimethyl-1,3-dioxane-4,6-dione (**7**, 38.74 g, 179.20 mmol) and allyl alcohol (57.4 mL, 537.60 mmol) in toluene (10.0 mL) was heated to 80 °C for 24 h. After cooling to room temperature the solvent was removed *in vacuo* and the residue was purified by bulb-to-bulb distillation to give 20.04 g (51.7%) of **13** as a yellow oil. ^1^H-NMR (200 MHz, CDCl_3_): δ (ppm) 3.36 (s, 2H), 3.65 (m, 2H), 3.96–3.99 (m, 2H), 4.04 (s, 2H), 4.22–4.27 (m, 2H), 5.27 (d, *J* = 19.1, 2H), 5.88 (m, 1H); ^13^C-NMR (50 MHz, CDCl_3_): δ (ppm) 45.6, 59.2, 64.3, 67.5, 71.9, 77.1, 117.1, 134.4, 166.9, 201.3; MS: *m/z* (%) 73 (23), 69 (37), 60 (23), 57 (41), 55 (60), 43 (100), 42 (17), 41 (87); HRMS: *m/z* calcd. for C_10_H_16_O_5_: 216.0998. Found: 216.1003.

*4-Methoxybenzyl 4-(3,4-difluorophenyl)-6-(methoxymethyl)-2-oxo-1,2,3,4-tetrahydropyrimidine-5-carboxylate* (**15**). To a well-stirred solution of 4-methoxybenzyl 4-methoxy-3-oxobutanoate (**8**, 330 mg, 1.31 mmol), 3,4-difluorobenzaldehyde (192 mg, 1.35 mmol) and urea (118 mg, 1.96 mmol) in THF (1.2 mL), Cu_2_O (19 mg, 0.13 mmol), glacial acetic acid (7.6 µL) and boron trifluoride diethyl etherate (0.2 mL, 240 mg, 1.69 mmol) were added. The resulting reaction mixture was heated under reflux for 8 h. After cooling to room temperature the mixture was poured onto ice (2 g) and NaHCO_3_ (200 mg). The resulting cloudy solution was filtered over Celite and washed with CH_2_Cl_2_ (5 mL). The biphasic solution was separated in a separatory funnel and the aqueous phase was washed with CH_2_Cl_2_ (3 × 30 mL). The combined organic layers were dried over Na_2_SO_4_, filtered and evaporated *in vacuo*. The product was partly purified by column chromatography (silica gel, eluent: EtOAc/MeOH 4:1) to give 315 mg (57.5%) of **15** as a yellow oil. The crude product was reacted without further purification in the next step.

*2-(Trimethylsilyl)ethyl 4-(3,4-difluorophenyl)-6-(methoxymethyl)-2-oxo-1,2,3,4-tetrahydropyrimidine-5-carboxylate* (**16**). To a well-stirred solution of 2-(trimethylsilyl)ethyl 4-methoxy-3-oxobutanoate (**9**, 2.35 g, 10.11 mmol), 3,4-difluorobenzaldehyde (1.48 mg, 10.41 mmol) and urea (910 mg, 15.17 mmol) in THF (8.7 mL), Cu_2_O (146 mg, 1.02 mmol), glacial acetic acid (59 µL) and boron trifluoride diethyl etherate (1.6 mL, 1.82 g, 12.80 mmol) were added. The resulting reaction mixture was heated under reflux for 8 h (TLC monitoring EtOAc/hexane 1:1). After cooling to room temperature the mixture was poured onto ice (15 g) and NaHCO_3_ (3 g). The resulting cloudy solution was filtered over Celite and washed with CH_2_Cl_2_ (12 mL). The biphasic solution was separated in a separatory funnel and the aqueous phase was washed with CH_2_Cl_2_ (3 × 10 mL). The combined organic layers were dried over Na_2_SO_4_, filtered and evaporated *in vacuo*. The crude product was purified by column chromatography (silica gel, eluent: CH_2_Cl_2_/MeOH 9:1) to give 2.05 mg (51.0%) of **16** as yellow oil. ^1^H-NMR (200 MHz, CDCl_3_): δ (ppm) −0.01 (s, 9H, 9c-Si(CH_3_)_3_), 0.84–0.93 (m, 2H, 9b-SiCH_2_), 3.42 (s, 3H, 7-OCH_3_), 4.05–4.14 (m, 2H, 9a-OCH_2_), 4.62 (s, 2H, 6-OCH_2_), 5.31 (s, 2H, 2a-NH), 6.96 (s, 1H, 3-CH), 7.01–7.15 (m, 3H, 11-CH, 14-CH, 15-CH), 7.65 (s, 1H, 1-NH); ^13^C-NMR (50 MHz, CDCl_3_): δ (ppm) −1.7 (9c-Si(CH_3_)_3_), 17.4 (9b-SiCH_2_), 54.3 (3-CH), 59.0 (7-OCH_3_), 62.3 (9a-OCH_2_), 68.5 (6-OCH_2_), 98.8 (4-C), 115.4/115.8 (11-CH), 117.1/117.6 (14-CH), 122.4/122.5 (15-CH), 140.5 (10-C), 147.1 (5-C), 152.5 (2-CO), 165.0 (8-COO); MS *m/z* (%): 398 (1), 370 (11), 355 (27), 323 (13), 293 (11), 281 (11), 257 (10), 253 (9), 225 (10), 185 (10), 167 (13), 84 (12), 75 (13), 73 (100), 45 (26); HRMS: Calcd. for C_18_H_24_F_2_N_2_O_4_SiNa [M + Na]^+^: 421.1371. Found: 421.1365.

*t-Butyl-4-(3,4-difluorophenyl)-6-(methoxymethyl)-2-oxo-1,2,3,4-tetrahydropyrimidine-5-carboxylate* (**17**). To a stirred mixture of *t-*butyl 4-methoxy-3-oxobutanoate **10** (3.00 g, 15.94 mmol), 3,4-difluorobenzaldehyde (1.8 mL, 2.33 g, 16.40 mmol) and urea (1.44 g, 23.98 mmol) in THF (14.0 mL), Cu_2_O (230 mg, 1.61 mmol) and CH_3_COOH were added at room temperature, followed by dropwise addition of boron trifluoride diethyl etherate (2.5 mL, 2.88 g, 20.20 mmol). The resulting mixture was stirred and refluxed for 8 h. After cooling to room temperature, the reaction mixture was poured into a mixture of ice (25 g) and NaHCO_3_ (5 g). The resulting mixture was filtered over Celite and washed with CH_2_Cl_2_ (20 mL). The organic phase was separated from the filtrate and the aqueous layer was extracted with CH_2_Cl_2_ (3 x 15 mL). The combined organic layers were dried over Na_2_SO_4_ and the solvent was evaporated *in vacuo* to give 4.92 g of raw product (a brown oil) that was purified *via* column chromatography (silica gel, eluent: CH_2_Cl_2_/MeOH 9:1) to give compound **17** as a yellow oil (1.74 g, 30.8%). ^1^H-NMR (200 MHz, CDCl_3_): δ (ppm) 1.32 (s, 9H, *t-*but-(CH_3_)_3_), 3.41 (s, 3H, 7-OCH_3_), 4.60 (s, 2H, 6-OCH_2_), 5.24 (s, 1H, 3-CH), 7.00–7.08 (m, 3H, 11-CH, 14-CH, 15-CH), 7.60 (s, 1H, NH); ^13^C-NMR (50 MHz, CDCl_3_): δ (ppm) 28.0 (*t-*but-(CH_3_)_3_), 54.7 (3-CH), 58.9 (7-OCH_3_), 68.5 (6-OCH_2_), 81.1 (*t-*but-C), 99.9 (4-C), 115.4/115.7 (11-CH), 117.1/117.4 (14-CH), 122.37/ 122.45/122.5/122.6 (15-CH), 140.7 (10-C), 146.3 (5-C), 152.5 (2-C), 164.03 (8-COO); MS: *m/z* (%) 354 (1), 298 (11), 265 (6), 221 (6), 185 (39), 121 (11), 71 (23), 70 (12), 69 (22), 57 (100), 55 (20), 43 (19), 41 (17); HRMS: Calcd. for C_17_H_20_F_2_N_2_O_4_Na [M + Na]^+^: 377.1289. Found: 377.1284.

*2-(t-Butyldiphenylsilyloxy)ethyl 4-(3,4-difluorophenyl)-6-(methoxymethyl)-2-oxo-1,2,3,4-tetrahydro-pyrimidine-5-carboxylate* (**19**). To a mixture of 2-hydroxyethyl 4-(3,4-difluorophenyl)-6-(methoxymethyl)-2-oxo-1,2,3,4-tetra­hydropyrimidine-5-carboxylate (**12a**, 0.78 g, 2.28 mmol), imidazole (155 mg, 2.28 mmol), and absolute CH_2_Cl_2_ (140 mL), *tert-*butylchlorodiphenylsilane (0.6 mL, 626 mg, 2.28mmol) dissolved in absolute CH_2_Cl_2_ was added dropwise at 0 °C. After stirring for 24 h at room temperature, the solvent was removed under reduced pressure and the residue was purified by column chromatography (silica gel, eluent: CH_2_Cl_2_/MeOH 9:1) to give 1.20 g (90.7%) of **19** as a colorless oil. ^1^H-NMR (200 MHz, CDCl_3_): δ (ppm) 1.06 (s, 9H, *t-*but-(CH_3_)_3_), 3.42 (s, 3H, 7-OCH_3_), 3.79–3.83 (m, 2H, 9b-OCH_2_), 4.06–4.29 (m, 2H, 9a-OCH_2_), 4.63 (s, 2H, 6-OCH_2_), 5.27 (d, 1H, *J* = 2.9 Hz, 3-CH), 6.59 (s, 1H, 2a-NH), 6.96–7.15 (m, 3H, 11-CH, 14-CH, 15-CH), 7.36–7.46 (m, 6H, 2′-(CH)_2_, 4′-(CH)_2_, 6′-(CH)_2_), 7.63–7.76 (m, 5H, 1-NH, 3′-(CH)_2_, 5′-(CH)_2_); ^13^C-NMR (50 MHz, CDCl_3_): δ (ppm) 19.1 (*t-*but-C), 26.7 (*t-*but-(CH_3_)_3_), 54.4 (3-CH), 59.0 (7-OCH_3_), 62.0 (9b-OCH_2_), 65.4 (9a-OCH_2_), 68.5 (6-OCH_2_), 98.4 (4-C), 115.3/115.7 (11-CH), 117.2/117.5 (14-CH), 122.4/122.5/122.6 (15-CH), 127.7 (3′-(CH)_2_, 5′-(CH)_2_), 129.8 (4′-(CH)_2_), 133.1 (1′-(C)_2_), 135.4 (2′-(CH)_2_, 6′-(CH)_2_), 140.4 (10-C), 147.5 (5-C), 152.3 (2-CO), 164.6 (8-COO); MS: *m/z* (%) 580 (1), 523 (18), 493 (4), 282 (15), 281 (100), 251 (51), 238 (12), 235 (12), 199 (18), 165 (18), 140 (30), 135 (12), 45 (10); HRMS: *m/z* calcd. for C_31_H_34_F_2_N_2_O_5_SiNa [M + Na]^+^: 603.2103. Found: 603.2121.

*Allyloxyethyl 4-(3,4-difluorophenyl)-6-(methoxymethyl)-2-oxo-1,2,3,4-tetrahydropyrimidine-5-carboxylate* (**20**). To a well-stirred solution of allyloxyethyl 4-methoxy-3-oxobutanoate (**13**, 20.04 g, 98.68 mmol), 3,4-difluorobenzaldehyde (9.8 mL, 12.55 g, 88.34 mmol) and urea (7.95 g, 132.51 mmol) in THF (113 mL), Cu_2_O (1.26 g, 8.83 mmol), glacial acetic acid (506 µL) and boron trifluoride diethyl etherate (114.8 mL, 130.17 g, 917.04 mmol) were added. The resulting reaction mixture was heated under reflux for 8 h. After cooling to room temperature the mixture was poured on a mixture of ice (125 g) and NaHCO_3_ (25 g). The resulting cloudy solution was filtered over Celite and washed with CH_2_Cl_2_ (5 mL). The biphasic solution was separated in a separatory funnel and the aqueous phase was washed with CH_2_Cl_2_ (3 × 40 mL). The combined organic layers were dried over Na_2_SO_4_, filtered and evaporated *in vacuo*. The crude product was purified by column chromatography (silica gel, eluent: EtOAc/petroleum ether 3:1) to give 9.00 g (24.4%) of **20** as yellow oil. ^1^H-NMR (200 MHz, CDCl_3_): δ (ppm) 3.43 (s, 3H), 3.94–4.23 (m, 6H), 4.63 (s, 2H), 5.16–5.29 (m, 2H), 5.76–5.96 (m, 1H), 6.52 (s, 1H), 7.04–7.21 (m, 3H), 7.66 (s, 1H); ^13^C-NMR (50 MHz, CDCl_3_): δ (ppm) 54.5, 59.1, 63.3, 67.7, 68.5, 71.9, 98.4, 115.6/115.9, 117.2, 117.4/117.6, 134.2, 147.6, 152.2, 164.5; MS: *m/z* (%) 382 (14), 280 (51), 269 (28), 222 (31), 221 (39), 167 (51), 45 (35), 41 (100); HRMS: *m/z* calcd for C_18_H_21_O_5_F_2_N_2_: 383.1419. Found: 383.1426; CHN: calcd for C_18_H_21_O_5_F_2_N_2_·H_2_O: C, 55.20; H, 5.16; N, 7.15. Found: C, 54.60; H, 5.10; N, 6.82.

*4-Methoxybenzyl 3-((3-bromopropyl)carbamoyl)-4-(3,4-difluorophenyl)-6-(methoxymethyl)-2-oxo-1,2,3,4-tetrahydropyrimidine-5-carboxylate* (**22**). To a solution of 4-methoxybenzyl 4-(3,4-difluorophenyl)-6-(methoxymethyl)-2-oxo-1,2,3,4-tetrahydropyrimidine-5-carboxylate (**15**, 252 mg, 0.60 mmol) and 4-nitrophenylchloroformate (425 mg, 2.11 mmol) in THF (7.5 mL), LiHMDS (1.7 mL, 284 mg, 1.70 mmol, 1 M in THF) was added dropwise at -78 °C. After 10 min the reaction was quenched with water (190 µL) and the mixture was allowed to warm to 0 °C. After addition of K_2_CO_3_ (333 mg, 2.41 mmol) and 3-aminopropylbromide hydrobromide (396 mg, 1.69 mmol) the reaction mixture was stirred at room temperature overnight. The yellow suspension was washed with saturated aqueous NaHCO_3_ and the biphasic solution was separated in a separatory funnel. The aqueous phase was extracted with Et_2_O (2 × 20 mL). The combined organic layers were dried over Na_2_SO_4_ and evaporated *in vacuo*. The crude product was purified by column chromatography (silica gel, eluent: CH_2_Cl_2_/MeOH 9:1) to give 70 mg (19.9%) of **22** as yellow oil. ^1^H-NMR (200 MHz, CDCl_3_): δ (ppm) 1.20-1.25 (m, 2H, 20-CH_2_), 1.99–2.08 (m, 2H, 19-CH_2_), 3.39–3.41 (m, 2H, 18-CH_2_), 3.43 (s, 3H, 7-OCH_3_), 3.91 (s, 3H, PMB-OCH_3_), 4.63 (s, 2H, 6-OCH_2_), 5.46 (s, 2H, PMB-OCH_2_), 6.59 (s, 1H, 3-CH), 6.79–6.85 (m, 3H, 11-CH, 14-CH, 15-CH), 6.99–7.14 (m, 4H, PMB-2′-CH, 3′-CH, 5′-CH, 6′-CH), 7.72 (s, 1H, 1-NH), 8.86 (t, 1H, J = 4.4 Hz, 17-NH); ^13^C-NMR (50 MHz, CDCl_3_): δ (ppm) 30.4 (20-CH_2_), 32.1 (19-CH_2_), 39.1 (18-CH_2_), 53.2 (3-CH), 55.2 (PMB-OCH_3_), 59.2 (7-OCH_3_), 66.7 (PMB-OCH_2_), 68.0 (6-OCH_2_), 106.9 (4-C), 113.9 (PMB-3′-CH, 5′-CH), 116.0/116.4 (11-CH), 117.1/117.4 (14-CH), 123.1 (15-CH), 130.3 (PMB-2′-CH, 6′-CH), 145.7 (2-CO), 153.3 (16-CO), 167.6/167.7 (8-COO), 5-C and 10-C not found; MS *m/z* (%): 325 (57), 294 (81), 279 (31), 265 (29), 222 (20), 137 (28), 121 (100), 45 (37); HRMS: Calcd. for C_25_H_26_F_2_N_3_O_6_Br [M − H]^-^: 580.0903. Found: 580.0895.

*2-(Trimethylsilyl)ethyl 3-((3-bromopropyl)carbamoyl)-4-(3,4-difluorophenyl)-6-(methoxymethyl)-2-oxo-1,2,3,4-tetrahydropyrimidine-5-carboxylate* (**23**). To a solution of 2-(trimethylsilyl)ethyl 4-(3,4-difluorophenyl)-6-(methoxymethyl)-2-oxo-1,2,3,4-tetrahydropyrimidine-5-carboxylate (**16**, 590 mg, 1.48 mmol) and 4-nitrophenylchloroformate (1.05 mg, 5.18 mmol) in THF (18.5 mL), LiHMDS (4.2 mL, 694 mg, 4.15 mmol, 1 M in THF) was added dropwise at ‒78 °C. After 10 min the reaction was quenched with water (460 µL) and the mixture was allowed to warm to 0 °C. After addition of K_2_CO_3_ (819 mg, 5.93 mmol) and 3-aminopropylbromide hydrobromide (973 mg, 4.44 mmol), the reaction mixture was stirred at room temperature overnight. The yellow suspension was washed with saturated aqueous NaHCO_3_ and the biphasic solution was separated in a separatory funnel. The aqueous phase was extracted with Et_2_O (2 × 20 mL). The combined organic layers were dried over Na_2_SO_4_ and evaporated *in vacuo*. The crude product was purified by column chromatography (silica gel, eluent: EtOAc/petroleum ether 1:1 and CH_2_Cl_2_/MeOH 9.5:0.5) to give 490 mg (59.0%) of **23** as yellow oil. ^1^H-NMR (200 MHz, CDCl_3_): δ (ppm) 0.14 (s, 9H, 9c-Si(CH_3_)_3_), 1.05–1.15 (m, 2H, 9b-SiCH_2_), 1.34–1.41 (m, 2H, 20-CH_2_), 2.18–2.28 (m, 19-CH_2_), 3.49–3.56 (m, 18-CH_2_), 3.59 (s, 3H, 7-OCH_3_), 4.18–4.35 (m, 2H, 9a-OCH_2_), 4.81 (s, 2H, 6-OCH_2_), 6.78 (s, 1H, 3-CH), 7.12–7.39 (m, 3H, 11-CH, 14-CH, 15-CH), 7.87 (s, 1H, 1-NH), 9.02 (t, 1H, J = 5.6 Hz, 17-NH); ^13^C-NMR (50 MHz, CDCl_3_): δ (ppm) −1.7 (9c-Si(CH_3_)_3_), 17.5 (9b-SiCH_2_), 30.4 (20-CH_2_), 32.2 (19-CH_2_), 39.1 (18-CH_2_), 53.0 (3-CH), 59.1 (7-OCH_3_), 63.1 (9a-OCH_2_), 68.1 (6-OCH_2_), 101.9 (4-C), 116.0/116.4 (11-CH), 117.1/117.4 (14-CH), 123.0/123.1 (15-CH), 137.4/137.5 (10-C), 147.4/147.6 (5-C), 152.5 (2-CO), 153.3 (16-CO), 164.3 (8-COO); MS *m/z* (%): 536 (100), 508 (4), 436 (15), 421 (9), 378 (71), 350 (40), 328 (3), 306 (5), 278 (17), 234 (14); HRMS: Calcd. for C_22_H_30_O_5_F_2_N_3_BrSiNa [M + Na]^+^: 584.1004. Found: 584.1008.

*t-Butyl-3-(3-bromopropylcarbamoyl)-4-(3,4-difluorophenyl)-6-(methoxymethyl)-2-oxo-1,2,3,4-tetra-hydropyrimidine-5-carboxylate* (**24**). To a solution of pyrimidinone **17** (1.65 g, 4.66 mmol) and *p*-nitrophenylchloroformate (3.29 g, 16.32 mmol) in THF (60.0 mL), LiHMDS (13.0 mL, 2.18 g, 13.03 mmol, 1 M in THF) was slowly added at −78 °C. After 10 min, the reaction was completed by addition of H_2_O (1.5 mL), warmed to 0 °C and neutralised with K_2_CO_3_ (2.57 g, 18.60 mmol). Thereafter, 3-aminopropylbromide hydrobromide (3.06 g, 13.98 mmol) was added and the reaction mixture was allowed to warm to room temperature overnight. The resulting yellow suspension was washed with NaHCO_3_ twice, the layers were separated and the aqueous layer was extracted with Et_2_O. The combined organic layers were dried over Na_2_SO_4_. After purification of the raw product (5.23 g, brown oil) *via* column chromatography (silica gel, eluent: CH_2_Cl_2_/MeOH 9.5:0.5), the bromide **24** could be obtained as a yellow oil (1.41 g, 58.2%). ^1^H-NMR (200 MHz, CDCl_3_): δ (ppm) 1.23 (t, 2H, *J* = 7.1 Hz, 20-CH_2_), 1.39 (s, 9H, *t-*but-(CH_3_)_3_), 2.04–2.21 (m, 2H, 19-CH_2_), 3.35–3.42 (m, 2H, 18-CH_2_), 3.45 (s, 3H, 7-OCH_3_), 4.65 (s, 2H, 6-OCH_2_), 6.54 (s, 1H, 3-CH), 7.02–7.23 (m, 3H, 11-CH, 14-CH, 15-H), 7.73 (s, 1H, 1-NH), 8.91 (t, 1H, *J* = 5.6 Hz, 17-NH); ^13^C-NMR (50 MHz, CDCl_3_): δ (ppm) 28.1 (*t-*but-(CH_3_)_3_), 30.5 (20-CH_2_Br), 32.1 (19-CH_2_), 39.1 (18-CH_2_), 53.6 (3-CH), 59.0 (7-OCH_3_), 68.1 (6-OCH_2_), 81.7 (*t-*but-C), 103.1 (4-C), 116.1/116.4 (11-CH), 117.0/117.3 (14-CH), 123.1 (15-CH), 137.8 (10-C), 147.5 (5-C), 152.5 (2-CO), 153.4 (16-CO), 163.3 (8-COO); MS: *m/z* (%) 518 (1), 353 (4), 322 (44), 297 (73), 279 (34), 266 (100), 265 (37), 221 (16), 185 (14), 167 (10), 57 (33), 41 (30); HRMS: Calcd. for C_21_H_26_F_2_N_3_O_5_BrNa [M + Na]^+^: 540.0922. Found: 540.0904.

*2-(t-Butyldiphenylsilyloxy)ethyl 3-((3-bromopropyl)carbamoyl)-4-(3,4-difluorophenyl)-6-(methoxy­methyl)-2-oxo-1,2,3,4-tetrahydropyrimidine-5-carboxylate* (**26**). To a solution of 2-(t-butyl-diphenylsilyloxy)ethyl 4-(3,4-difluorophenyl)-6-(methoxymethyl)-2-oxo-1,2,3,4-tetrahydro­pyrimidine-5-carboxylate (**19**, 1.10 g, 1.89 mmol) and 4-nitrophenylchloroformate (1.34 g, 6.65 mmol) in THF (24.0 mL), LiHMDS (5.3 mL, 892 mg, 5.33 mmol, 1 M in THF) was added dropwise at −78 °C. After 10 min the reaction was quenched with water (9.0 mL) and the mixture was allowed to warm to 0 °C. After addition of K_2_CO_3_ (1.05 g, 7.60 mmol) and 3-aminopropylbromide hydrobromide (1.25 g, 5.71 mmol) the reaction mixture was stirred at room temperature overnight. The yellow suspension was washed with saturated aqueous NaHCO_3_ and the biphasic solution was separated in a separatory funnel. The aqueous phase was extracted with Et_2_O. The combined organic layers were dried over Na_2_SO_4_ and evaporated *in vacuo*. The crude product was purified by column chromatography (silica gel, eluent: EtOAc/petroleum ether 1:1) to give 1.16 g (82.2%) of **26** as a yellow oil. ^1^H-NMR (200 MHz, CDCl_3_): δ (ppm) 0.96 (s, 9H, t-but-(CH_3_)_3_), 1.17–1.24 (m, 2H, 20-CH_2_), 2.01–2.11 (m, 2H, 19-CH_2_), 3.32–3.51 (m, 5H, 18-CH_2_, 7-OCH_3_), 3.74–3.79 (m, 2H, 9b-OCH_2_), 4.12–4.24 (m, 2H, 9a-OCH_2_), 4.61 (s, 2H, 6-OCH_2_), 6.64 (s, 1H, 3-CH), 6.78–7.20 (m, 3H, 11-CH, 14-CH, 15-CH), 7.28–7.37 (m, 6H, 2′-(CH)_2_, 4′-(CH)_2_, 6′-(CH)_2_), 7.54–7.58 (m, 4H, 3′-(CH)_2_, 5′-(CH)_2_), 7.73 (s, 1H, 1-NH), 8.86 (t, J = 5.7 Hz, 17-NH); ^13^C-NMR (50 MHz, CDCl_3_): δ (ppm) 19.0 (t-but-C), 26.6 (t-but-(CH_3_)_3_), 30.4 (20-CH_2_), 32.1 (19-CH_2_), 39.1 (18-CH_2_), 53.2 (3-CH), 59.1 (7-OCH_3_), 61.8 (9b-OCH_2_), 65.7 (9a-OCH_2_), 68.1 (6-OCH_2_), 101.6 (4-C), 115.5/115.8/116.2 (11-CH), 117.2/117.5 (14-CH), 123.0/123.1/ 123.2 (15-CH), 127.7 (3′-(CH)_2_, 5′-(CH)_2_), 129.8 (4′-(CH)_2_), 133.1 (1′-(C)_2_), 135.4 (2′-(CH)_2_, 6′-(CH)_2_), 137.4 (10-C), 145.9 (5-C), 152.5 (2-CO), 153.3 (16-CO), 164.6 (8-COO); MS: *m/z* (%) 768 (53), 686 (2), 603 (100), 560 (47), 540 (16), 460 (1), 238 (2); HRMS: *m/z* calcd for C_35_H_40_F_2_N_3_O_6_BrSiNa [M + Na]^+^: 766.1736. Found: 766.1728.

*Allyloxyethyl 3-((3-bromopropyl)carbamoyl)-4-(3,4-difluorophenyl)-6-(methoxymethyl)-2-oxo-1,2,3,4-tetrahydropyrimidine-5-carboxylate* (**27**). To a solution of allyloxyethyl 4-(3,4-difluorophenyl)-6-(methoxymethyl)-2-oxo-1,2,3,4-tetrahydropyrimidine-5-carboxylate (**20**, 9.00 g, 23.56 mmol) and 4-nitrophenylchloroformate (16.65 g, 82.60 mmol) in THF (300.0 mL), LiHMDS (66.08 mL, 11.06 g, 66.08 mmol, 1 M in THF) was added dropwise at −78 °C. After 10 min the reaction was quenched with water (9.0 mL) and the mixture was allowed to warm to 0 °C. After addition of K_2_CO_3_ (13.03 g, 94.40 mmol) and 3-aminopropylbromide hydrobromide (15.50 g, 70.80 mmol) the reaction mixture was stirred at room temperature overnight. The yellow suspension was washed with saturated aqueous NaHCO_3_ and the biphasic solution was separated in a separatory funnel. The aqueous phase was extracted with Et_2_O. The combined organic layers were dried over Na_2_SO_4_ and evaporated *in vacuo*. The crude product was purified by column chromatography (silica gel, eluent: CH_2_Cl_2_/MeOH 10:1) to give 6.60 g (51.3%) of **27** as a yellow oil. ^1^H-NMR (200 MHz, CDCl_3_): δ (ppm) 2.02–2.08 (m, 2H), 3.36–3.47 (m, 5H), 3.60–3.62 (m, 2H), 3.94–4.29 (m, 6H), 4.65 (s, 1H), 5.15–5.28 (m, 2H), 5.75–5.94 (m, 1H), 6.64 (s, 1H), 7.02–7.25 (m, 3H), 7.77 (s, 1H), 8.89 (t, J = 5.6 Hz, 1H); ^13^C-NMR (50 MHz, CDCl_3_): δ (ppm) 30.4, 32.1, 39.1, 59.1, 63.7, 67.6, 68.1, 71.9, 101.5, 116.0/116.4, 117.1, 117.3/117.4, 122.9, 134.2, 137.8, 146.0, 152.5, 153.2, 163.9; MS: *m/z* (%) 546 (2), 381 (64), 350 (52), 311 (30), 280 (48), 279 (100), 249 (33), 222 (47), 220 (32), 167 (29), 41 (91); HRMS: *m/z* calcd. for C_22_H_26_O_6_BrF_2_N_3_H: 548.1035. Found (M+1)^+^: 548.1044; CHN: Calcd. for C_22_H_26_O_6_BrF_2_N_3_H·H_2_O: C, 47.58; H, 4.73; N, 7.57. Found: C, 47.47; H, 4.81; N, 7.28.

*4-Methoxybenzyl 3-((3-(4-(3-acetamidophenyl)piperidin-1-yl)propyl)carbamoyl-4-(3,4-difluoro-phenyl)-6-(methoxymethyl)-2-oxo-1,2,3,4-tetrahydropyrimidine-5-carboxylate* (**29**). Under an argon atmosphere a mixture of N-(3-(piperidin-4-yl)phenyl)acetamide (**28**, 109 mg, 0.50 mmol), 4-methoxybenzyl 3-((3-bromopropyl)carbamoyl)-4-(3,4-difluorophenyl)-6-(methoxymethyl)-2-oxo-1,2,3,4-tetrahydropyrimidine-5-carboxylate (**22**, 190 mg, 0.33 mmol) and K_2_CO_3_ (480 mg, 3.40 mmol) in ACN (12.6 mL) was stirred at 35 °C for 37 h. The resulting yellow suspension was filtered and the filtration residue was washed with EtOAc. After removal of the solvent *in vacuo* the obtained oily residue was dissolved in EtOAc (10 mL) and the organic phase was washed with saturated aqueous NaHCO_3_ (2 × 8 mL). The aqueous phase was washed with EtOAc (2 × 10 mL) and the combined organic layers were dried over Na_2_SO_4_, filtered and evaporated *in vacuo*. The obtained residue was purified by column chromatography (silica gel, eluent: CH_2_Cl_2_/MeOH 10:1) to give 75 mg (30.3%) of **29** as yellow oil. ^1^H-NMR (200 MHz, CDCl_3_): δ (ppm) 1.67–1.79 (m, 6H, 19-CH_2_, 22,22′-(CH_2_)_2_), 2.00–2.13 and 3.08–3.13 (m, 7H, 21,21′-(CH_2_)_2_, 32-CH_3_), 2.48–2.51 (m, 3H, 20-CH_2_, 23-CH), 3.29–3.41 (m, 5H, 7-OCH_3_, 18-CH_2_), 3.78 (s, 3H, PMB-OCH_3_), 4.65 (d, 2H, J = 2.6 Hz, 6-OCH_2_), 6.62 (d, 1H, J = 6.2 Hz, 3-CH), 6.81–7.22 (m, 10H, 11-CH, 14-CH, 15-CH, 27-CH, 28-CH, 29-CH, 2′-CH, 3′-CH, 4′-CH, 5′-CH), 7.39 (25-CH), 7.97 (br s, 1H, 30-NH), 8.15 (s, 1H, 1-NH), 8.96–8.98 (m, 1H, 17-NH); ^13^C-NMR (50 MHz, CDCl_3_): δ (ppm) 24.3 (32-CH_3_), 25.8 (19-CH_2_), 32.2 (22,22′-(CH_2_)_2_), 39.2 (18-CH_2_), 42.0 (23-CH), 53.2 (3-CH), 53.9 (21,21′-(CH_2_)_2_), 55.2 (PMB-OCH_3_), 56.1 (20-CH_2_), 59.0 (7-OCH_3_), 66.3 (PMB-OCH_2_), 68.0 (6-OCH_2_), 101.9 (4-C), 113.9 (3′-CH, 5′-CH), 116.0/116.3 (11-CH), 117.0/117.4 (14-CH), 117.8 (27-CH), 118.0 (25-CH), 122.7 (29-CH), 123.0 (15-CH), 127.4 (1′-C), 128.8 (28-CH), 130.0 (2′-CCH, 6′-CH), 137.8 (10-C), 138.4 (26-C), 145.9 (5-C), 146.5 (24-C), 152.3 (2-CO), 153.2 (16-CO), 164.1 (8-COO), 168.8 (31-CON), 4’-C not found; MS *m/z* (%): 301 (8), 231 (82), 213 (17), 167 (8), 153 (10), 121 (100), 95 (12), 70 (16), 57 (20); HRMS: Calcd. for C_38_H_43_F_2_N_5_O_7_: 720.3209. Found: 720.3216.

*2-(Trimethylsilyl)ethyl 3-((3-(4-(3-acetamidophenyl)piperidin-1-yl)propyl-carbamoyl)-4-(3,4-difluoro-phenyl)-6-methoxymethyl)-2-oxo-1,2,3,4-tetrahydropyrimidine-5-carboxylate* (**30**). Under an argon atmosphere a mixture of *N*-(3-(piperidin-4-yl)phenyl)acetamide (**28**, 251 mg, 1.15 mmol), 2-(trimethylsilyl)ethyl 3-((3-bromopropyl)carbamoyl)-4-(3,4-difluorophenyl)-6-(methoxymethyl)-2-oxo-1,2,3,4-tetrahydropyrimidine-5-carboxylate (**23**, 420 mg, 0.75 mmol) and K_2_CO_3_ (1.09 g, 7.89 mmol) in ACN (28.0 mL) was stirred at 35 °C for 37 h. The resulting yellow suspension was filtered and the filtration residue was washed with EtOAc. After removal of the solvent *in vacuo* the obtained oily residue was dissolved in EtOAc (20 mL) and the organic phase was washed with saturated aqueous NaHCO_3_ (2 × 15 mL). The aqueous phase was washed with EtOAc (2 × 20 mL) and the combined organic layers were dried over Na_2_SO_4_, filtered and evaporated *in vacuo*. The obtained residue was purified by column chromatography (silica gel, eluent: CH_2_Cl_2_/MeOH 9:1 + 0.5% TEA) to give 240 mg (45.8%) of **30** as a yellow oil. ^1^H-NMR (200 MHz, CDCl_3_): δ (ppm) −0.02 (s, 9H, 9c-Si(CH_3_)_3_), 0.93–1.02 (m, 2H, 9b-SiCH_2_), 1.76 (m, 6H, 19-CH_2_, 22,22′-(CH_2_)_2_), 1.96–2.08 and 2.98–3.03 (m, 4H, 21,21′-(CH_2_)_2_), 2.15 (s, 3H, 32-CH_3_), 2.43 (t, 3H, J = 6.8 Hz, 20-CH_2_, 23-CH), 3.31–3.41 (m, 2H, 18-CH_2_), 3.44 (s, 3H, 7-OCH_3_), 4.15–4.24 (m, 2H, 9a-OCH_2_), 4.68 (s, 2H, 6-OCH_2_), 6.69 (s, 1H, 3-CH), 6.93 (d, 1H, J = 7.6 Hz, 29-CH), 7.03–7.35 (m, 5H, 11-CH, 14-CH, 15-CH, 27-CH, 28-CH), 7.49 (s, 1H, 25-CH), 8.05 (s, 1H, 30-NH), 8.25 (s, 1H, 1-NH), 9.02 (t, 1H, J = 5.1 Hz, 17-NH); ^13^C-NMR (50 MHz, CDCl_3_): δ (ppm) −1.8 (9c-Si(CH_3_)_3_), 17.3 (9b-SiCH_2_), 24.2 (32-CH_3_), 26.2 (19-CH_2_), 32.7 (22,22′-(CH_2_)_2_), 39.4 (18-CH_2_), 42.4 (23-CH), 52.9 (3-CH), 54.1 (21,21′-(CH_2_)_2_), 56.5 (20-CH_2_), 58.9 (7-OCH_3_), 63.0 (9a-OCH_2_), 67.9 (6-OCH_2_), 102.0 (4-C), 115.9/116.2 (11-CH), 116.9/117.3 (14-CH), 117.6 (27-CH), 118.2 (25-CH), 122.5 (29-CH), 122.8/122.9 (15-CH), 128.6 (28-CH), 137.6 (10-C), 138.2 (26-C), 145.8 (5-C), 147.0 (24-C), 152.3 (2-CO), 153.1 (16-CO), 164.3 (8-COO), 168.7 (31-CON); MS *m/z* (%): 700 (26), 600 (2), 345 (8), 259 (4), 231 (4); HRMS: Calcd. for C_35_H_48_F_2_N_5_O_6_Si [M + H]^+^: 700.3342. Found: 700.3343.

*t-Butyl-3-(3-(4-(3-acetamidophenyl)piperidin-1-yl)propylcarbamoyl)-4-(3,4-difluorophenyl)-6-(methoxymethyl)-2-oxo-1,2,3,4-tetrahydropyrimidine-5-carboxylate* (**31**). To a solution of N-(3-(piperidin-4-yl)phenyl)acetamide (**28**, 140 mg, 0.64 mmol) in ACN (16.0 mL), bromide **24** (215 mg, 0.41 mmol) and K_2_CO_3_ (605 mg, 4.38 mmol) were added under argon atmosphere and the mixture was stirred at 35 °C for 37 h. The yellow slurry was filtered, washed with EtOAc and the filtrate was evaporated to dryness. The oily residue was dissolved in EtOAc and washed twice with saturated NaHCO_3_. Then the aqueous phase was extracted with EtOAc. The combined organic layers were dried over Na_2_SO_4_ and evaporated *in vacuo* prior to purification by column chromatography (silica gel, eluent: CH_2_Cl_2_/MeOH 10:1). Product **31** was obtained as a yellow-brownish oil (133 mg, 49.4%). ^1^H-NMR (200 MHz, CDCl_3_): δ (ppm) 1.41 (t-but-(CH_3_)_3_), 1.74–1.78 (m, 6H, 19-CH_2_, 22,22′-(CH_2_)_2_), 1.94–2.07 and 2.98–3.03 (m, 4H, 21,21′-(CH_2_)_2_), 2.15 (s, 3H, 32-CH_3_), 2.37–2.44 (m, 3H, 20-CH_2_, 23-CH), 3.32–3.39 (m, 2H, 18-CH_2_), 3.44 (s, 3H, 7-CH_3_), 4.66 (s, 2H, 6-CH_2_), 6.58 (s, 1H, 3-CH), 6.94 (d, 1H, J = 7.3 Hz, 29-CH), 7.03–7.34 (m, 6H, 11-CH, 14-CH, 15-CH, 25-CH, 27-CH, 28-CH), 7.45 (s, 1H, 30-NH), 7.82 (s, 1H, 1-NH), 9.00 (t, 1H, J = 5.2 Hz, 17-NH); ^13^C-NMR (50 MHz, CDCl_3_): δ (ppm) 24.4 (32-CH_3_), 26.4 (19-CH_2_), 28.1 (t-but-(CH_3_)_3_), 32.9 (22,22′-(CH_2_)_2_), 39.5 (18-CH_2_), 42.5 (23-CH), 53.5 (3-CH), 54.3 (21,21′-(CH_2_)_2_), 56.5 (20-CH_2_), 59.0 (7-OCH_3_), 68.0 (6-OCH_2_), 103.3 (4-C), 116.0/116.4 (11-CH), 116.9/117.3 (14-CH), 117.6 (27-CH), 118.3 (25-CH), 122.7 (29-CH), 123.0/123.09/123.14/123.2 (15-CH), 128.8 (28-CH), 138.0 (10-C), 138.1 (26-C), 144.8 (5-C), 147.2 (24-C), 152.3 (2-CO), 153.3 (16-CO), 163.5 (8-COO), 168.5 (31-CON); MS: *m/z* (%) 679 (100, [M + Na]^+^), 657 (20, [M + H]^+^), 621 (10), 601 (17), 324 (23), 302 (49); HRMS: Calcd. for C_34_H_44_F_2_N_5_O_6_ [M + H]^+^: 656.3260. Found: 656.3277; IR: (ν) (cm^−1^) 3304, 2925, 1709, 1608, 1516, 1392, 1366, 1278, 1230, 1164, 1119, 1080, 772, 732, 701.

*2-(t-Butyldiphenylsilyloxy)ethyl 3-((3-(4-(3-acetamidophenyl)piperidin-1-yl)propyl)carbamoyl-4-(3,4-difluorophenyl)-6-(methoxymethyl)-2-oxo-1,2,3,4-tetrahydropyrimidine-5-carboxylate* (**33**). Under an argon atmosphere a mixture of N-(3-(piperidin-4-yl)phenyl)acetamide (**28**) (239 mg, 1.09 mmol), 2-(t-butyldiphenylsilyloxy)ethyl 3-((3-bromopropyl)carbamoyl)-4-(3,4-difluorophenyl)-6(methoxymethyl)- 2-oxo-1,2,3,4-tetrahydropyrimidine-5-carboxylate (**26**, 775 mg, 1.04 mmol) and K_2_CO_3_ (1.52 g, 11.00 mmol) in ACN (40 mL) was stirred at 35 °C for 37 h. The resulting yellow suspension was filtered and the filtration residue was washed with EtOAc. After removal of the solvent *in vacuo* the obtained oily residue was dissolved in EtOAc and the organic phase was washed with saturated aqueous NaHCO_3_. The aqueous phase was washed with EtOAc and the combined organic layers were dried over Na_2_SO_4_, filtered and evaporated *in vacuo*. The obtained residue was purified by column chromatography (silica gel, eluent: CH_2_Cl_2_/MeOH 9:1) to give 428mg (47.7%) of **33** as a yellow oil. ^1^H-NMR (200 MHz, CDCl_3_): δ (ppm) 1.01 (s, 9H, t-but-(CH_3_)_3_), 1.25–1.32 (m, 2H, 20-CH_2_), 1.74–1.94 (m, 6H, 19-CH_2_, 22,22′-(CH_2_)_2_), 2.01–2.09 and 3.09–3.14 (m, 4H, 21,21′-(CH_2_)_2_), 2.14 (s, 3H, 32-CH_3_), 2.48–2.60 (m, 3H, 20-CH_2_, 23-CH), 3.30-3.41 (m, 5H, 18-CH_2_, 7-OCH_3_), 3.79–3.84 (m, 2H, 9b-OCH_2_), 4.13–4.32 (m, 2H, 9a-OCH_2_), 4.65 (s, 2H, 6-OCH_2_), 6.69 (s, 1H, 3-CH), 6.90-7.26 (m, 7H, 11-CH, 14-CH, 15-CH, 25-CH, 27-CH, 28-CH, 29-CH), 7.33–7.42 (m, 6H, 2′-(CH)_2_, 4′-(CH)_2_, 6′-(CH)_2_), 7.59–7.63 (m, 5H, 3′-(CH)_2_, 5′-(CH)_2_, 30-NH), 8.15 (s, 1H, 1-NH), 8.86 (t, J = 5.7 Hz, 17-NH); ^13^C-NMR (50 MHz, CDCl_3_): δ (ppm) 19.0 (t-but-C), 24.3 (32-CH_3_), 25.8 (19-CH_2_), 26.6 (t-but-(CH_3_)_3_), 32.1 (22,22′-(CCH_2_)_2_), 39.2 (18-CH_2_), 42.0 (23-CH), 53.1 (3-CH), 53.8 (21,21′-(CH_2_)_2_), 56.0 (20-CH_2_), 59.0 (7-OCH_3_), 61.8 (9b-OCH_2_), 65.6 (9a-OCH_2_), 68.0 (6-OCH_2_), 101.7 (4-C), 115.8/116.2 (11-CH), 117.1/117.4 (14-CH), 117.8 (27-CH), 118.0 (25-CH), 122.9/ 122.8/123.0/123.1 (15-CH), 127.7 (3′-(CH)_2_, 5′-(CH)_2_), 128.8 (28-CH), 129.7 (4′-(CH)_2_), 133.0 (1′-(C)_2_), 135.4 (2′-(CH)_2_, 6′-(CH)_2_), 137.6 (10-C), 138.3 (26-C), 146.1 (5-C), 146.4 (24-C), 152.3 (2-CO), 153.1 (16-CO), 164.1 (8-COO), 168.7 (31-CON); MS: *m/z* (%) 883 (1), 523 (12), 281 (100), 231 (44), 199 (18), 168 (19), 140 (16), 70 (30), 57 (65), 56 (33), 45 (44), 43 (60), 41 (28); HRMS: *m/z* calcd. for C_48_H_57_F_2_N_5_O_7_Si: 882.4074. Found: 882.4087.

*Allyloxyethyl 3-((3-(4-(3-acetamidophenyl)piperidin-1-yl)propyl)carbamoyl-4-(3,4-difluorophenyl)-6-(methoxymethyl)-2-oxo-1,2,3,4-tetrahydropyrimidine-5-carboxylate* (**34**). Under an argon atmosphere a mixture of N-(3-(piperidin-4-yl)phenyl)acetamide (**28**, 4.04 mg, 18.47 µmol), allyloxyethyl 3-((3-bromopropyl)carbamoyl)-4-(3,4-difluorophenyl)-6-(methoxymethyl)-2-oxo-1,2,3,4-tetrahydropyrimidine-5-carboxylate (**27**, 6.60 g, 12.08 mmol) and K_2_CO_3_ (17.58 g, 127.20 mmol) in ACN (12.6 mL) was stirred at 35 °C for 37 h. The resulting yellow suspension was filtered and the filtration residue was washed with EtOAc. After removal of the solvent *in vacuo* the obtained oily residue was dissolved in EtOAc and the organic phase was washed with saturated aqueous NaHCO_3_. The aqueous phase was washed with EtOAc and the combined organic layers were dried over Na_2_SO_4_, filtered and evaporated *in vacuo*. The obtained residue was purified by column chromatography (silica gel, eluent: CH_2_Cl_2_/MeOH 9:1) to give 2.66 g (32.2%) of **34** as a yellow oil. ^1^H-NMR (200 MHz, CDCl_3_): δ (ppm) 1.72–1.81 (m, 6H), 1.99, 2.99 (m, 4H), 2.11 (s, 3H), 2.39 (m, 2H), 2.42 (m, 1H), 3.29, 3.39 (m, 2H), 3.37 (s, 3H), 3.55–3.60 (m, 2H), 3.92, 3.94 (d, J = 5.6 Hz, 2H), 4.17, 4.29 (m, 2H), 4.63 (m, 2H), 5.12–5.26 (m, 2H), 5.81 (m, 1H), 6.65 (s, 1H), 6.90 (d, J = 7.44, 1H), 6.98–7.19 (m, 5H), 7.25 (s, 1H), 7.44 (s, 1H), 7.95 (s, 1H), 8.98 (t, J = 5.3 Hz, 1H); ^13^C-NMR (50 MHz, CDCl_3_): δ (ppm) 24.4, 26.3, 32.8, 39.5, 42.5, 53.1, 54.2, 56.5, 58.9, 63.6, 67.6, 67.9, 71.9, 101.7, 115.9/116.3, 116.9, 117.2/117.3, 117.6, 118.2, 112.7, 122.8, 128.7, 134.2, 137.8, 138.2, 146.2, 147.16, 152.3, 153. 0, 164.0, 168.6; MS: *m/z* (%) 280 (33), 231 (100), 221 (21), 167 (34), 70 (26), 45 (21), 44 (21), 43 (54), 42 (31), 41 (57); HRMS: *m/z* calcd. for C_35_H_43_O_7_F_2_N_5_H: 684.3209. Found (M+1)^+^: 684.3195; CHN: calcd. for C_35_H_43_O_7_F_2_N_5_H•H_2_O: C, 59.90; H, 6.19; N, 9.98. Found: C, 59.09; H, 6.35; N, 9.69.

*(E)-2-Prop-1-en-1-yloxy)ethyl 3-((3-(4-(3-acetamidophenyl)piperidin-1-yl)propyl)carbamoyl-4-(3,4-difluorophenyl)-6-(methoxymethyl)-2-oxo-1,2,3,4-tetrahydropyrimidine-5-carboxylate* (**34a**). To a solution of allyloxyethyl 3-((3-(4-(3-acetamidophenyl)piperidin-1-yl)propyl)carbamoyl-4-(3,4-difluorophenyl)-6-(methoxymethyl)-2-oxo-1,2,3,4-tetrahydropyrimidine-5-carboxylate (**34**, 1.45 g, 2.12 mmol) and DABCO (1.00 g, 8.90 mmol) in EtOH (20 mL, 90%), RhCl(PPh_3_)_3_ (0.51 g, 0.55 mmol) was added. The reaction mixture was stirred for 15 min, cooled to room temperature, and then quenched with water. The aqueous phase was extracted with Et_2_O and dried with Na_2_SO_4_. After evaporation of the solvent, the crude product **34a** was used for the next step without further purification.

*2-Hydroxyethyl 3-((3-(4-(3-acetamidophenyl)piperidine-1-yl)propyl)carbamoyl-4-(3,4-difluoro­phenyl)-6-(methoxymethyl)-2-oxo-1,2,3,4-tetrahydropyrimidine-5-carboxylate* (**35**). Method 1(from **32**): To a solution of OsO_4_ (2.5% in *t*-butanol, 0.5 mL, 0.04 mmol), *N*-methyl morpholine *N*-oxide monohydrate (46 mg, 0.39 mmol), H_2_O (0.8 mL), acetone (0.3 mL), and *t*-butanol (0.5 mL), allyl ester **32** (250 mg, 0.39 mmol) dissolved in dioxane (0.5 mL) was added dropwise. After stirring at room temperature overnight, the mixture was treated with celite (62 mg) and NaHSO_3_ (5 mg) and filtered over celite. After evaporation of the solvent, the crude 2,3-dihydroxypropyl ester **32a** obtained was used for the next step.

To a solution of **32a** (267 mg) in CH_2_Cl_2_ (0.6 mL), a solution of NaIO_4_ (93 mg, 0.44 mmol) in H_2_O (0.6 mL) was added. The two-layered mixture was stirred for 3–4 h, followed by separation of the organic layer. After evaporation of the solvent and drying *in vacuo*, the crude residue **32b** was used in the next step without any further purification.

To a solution of **32b** (88 mg) in MeOH (3.0 mL), NaBH_4_ (6.0 mg, 0.15 mmol) was added in portions under stirring, followed by stirring for another 45 min. The reaction was quenched with water, and the mixture extracted three times with Et_2_O. The organic layer was washed with water. After evaporation of the solvent the crude product was purified *via* column chromatography (silica gel, eluent: CH_2_Cl_2_/MeOH 10:1) to give 12 mg of **35** (13.3%) as a yellow oil.

Method 2(from **33**): To a solution of 2-(*t-*butyldiphenylsilyloxy)ethyl 3-((3-(4-(3-acetamidophenyl)piperidin-1-yl) propyl)carbamoyl-4-(3,4-difluorophenyl)-6-(methoxymethyl)-2-oxo-1,2,3,4-tetrahydropyrimidine-5-carboxylate (**33**) in THF (6.5 mL), TBAF (0.5 mL, 452 mg, 1.73, 1M in THF) was added dropwise. After stirring for 1.5 h at room temperature, the reaction was quenched with water (0.3 mL) and evaporated to dryness. The crude product was purified by column chromatography (reversed-phase silica gel, eluent: ACN/H_2_O 5:1 and silica gel, eluent: EtOAc/MeOH 10:1) to give 126 mg (40.3%) of **35** as a yellow oil.

Method 3(from **34a**): A solution of HgCl_2_ (1.02 g, 3.76 mmol) in acetone/H_2_O (10:1, 10 mL) was added dropwise over a period of 3 min under stirring to a solution of (*E*)-2-(prop-1-en-1-yloxy)ethyl 3-((3-(4-(3-acetamidophenyl)piperidin-1-yl)propyl)carbamoyl)-4-(3,4-difluorophenyl)-6-(methoxy-methyl)-2-oxo-1,2,3,4-tetrahydropyrimidine-5-carboxylate **34a** and HgO (1.02 g, 4.71 mmol) in acetone/H_2_O (10:1, 30 mL). After completion of the reaction (TLC-monitoring), HgO was removed by filtration over celite and the product was evaporated to dryness. The residue was purified by column chromatography (silica gel, eluent: CH_2_Cl_2_/MeOH 10:1) where part of the educt **34a** could be recovered and used in another reaction again. The product was obtained in good yield (677 mg, 70.8%). ^1^H-NMR (200 MHz, CDCl_3_): δ (ppm) 1.74–1.85 (m, 6H, 19-CH_2_, 22,22′-(CH_2_)_2_), 2.02–2.09 and 3.01–3.06 (m, 4H, 21,21′-(CH_2_)_2_), 2.09 (s, 3H, 32-CH_3_), 2.42–2.45 (m, 3H, 20-CH_2_, 23-CH), 3.27–3.31 (m, 2H, 18-CH_2_), 3.39 (s, 3H, 7-OCH_3_), 3.74 (t, 2H, J = 4.4 Hz, 9b-CH_2_OH), 4.10-4.20 (m, 2H, 9a-OCH_2_), 4.62 (s, 2H, 6-OCH_2_), 6.62 (s, 1H, 3-CH), 6.86 (d, 1H, J = 7.5 Hz, 29-CH), 6.94–7.23 (m, 5H, 11-CH, 14-CH, 15-CH, 27-CH, 28-CH), 7.33–7.39 (m, 2H, 25-CH, 30-NH), 8.53 (s, 1H, 1-NH), 8.99 (t, 1H, J = 5.4 Hz, 17-NH); ^13^C-NMR (50 MHz, CDCl_3_): δ (ppm) 24.1 (32-CH_3_), 25.8 (19-CH_2_), 32.2 (22,22′-(CH_2_)_2_), 39.2 (18-CH_2_), 42.0 (23-CH), 53.0 (3-CH), 53.9 (21,21′-(CH_2_)_2_), 56.1 (20-CH_2_), 58.9 (7-OCH_3_), 60.3 (9b-CH_2_OH), 66.2 (9a-OCH_2_), 67.9 (6-OCH_2_), 101.6 (4-C), 115.8/116.1 (11-CH), 117.1/117.4 (14-CH), 117.8 (27-CH), 118.0 (25-CH), 122.6 (29-CH), 122.7/122.8 (15-CH), 128.7 (28-CH), 137.6 (10-C), 138.3 (26-C), 146.4 (5-C), 147.4 (24-C), 152.2 (2-CO), 153.2 (16-CO), 164.1 (8-COO), 169.0 (31-CON); MS: *m/z* (%) 644 (32), 345 (16), 302 (100), 259 (17), 231 (20), 160 (4), 114 (5); HRMS: Calcd. for C_32_H_40_F_2_N_5_O_7_ [M + H]^+^: 644.2896. Found: 644.2902.

3*-Hydroxypropyl-3-(3-(4-(3-acetamidophenyl)piperidin-1-yl)propylcarbamoyl)-4-(3,4-difluorophenyl)-6-(methoxymethyl)-2-oxo-1,2,3,4-tetrahydropyrimidine-5-carboxylate* (**36**). A solution of BH_3_-THF (1M, 0.31 mmol, 0.3 mL) was added dropwise to a solution of allyl ester **32** (131 mg, 0.21 mmol) in THF. The mixture was stirred at room temperature for 30 min prior to elimination of excess hydride ions via addition of water (0.4 mL). 3M NaOH solution (0.2 mL) and H_2_O_2_ solution (30%, 0.2 mL) were then added to the mixture. The stirred solution was heated to 60 °C for 2 h. Thereafter, the solution was cooled to room temperature and the solvent evaporated *in vacuo*. H_2_O was added and the reaction mixture was washed several times with CH_2_Cl_2_. The combined organic layers were dried over Na_2_SO_4_ and evaporated *in vacuo*. The intermediate product (108 mg) gave after purification by column chromatography (silica gel, eluent: CH_2_Cl_2_/MeOH 10:1) 43 mg of educt **32** and 23 mg of product **36** (26.1%). ^1^H-NMR (500 MHz, CDCl_3_): δ (ppm) 1.81–1.95 (22,22′-(CH_2_)_2_), 1.82 (m, 9b-CH_2_), 1.84 (m, 19-CH_2_), 2.16 and 3.13 (21,21′-(CH_2_)_2_), 2.17 (s, 32-CH_3_), 2.50 (m, 23-CH), 2.54 (m, 20-CH_2_), 3.33 and 3.41 (m, 18-CH_2_), 3.45 (s, 7-CH_3_), 3.52–3.64 (m, 9c-CH_2_), 4.22–4.31 (m, 9a-CH_2_), 4.67 (s, 6-CH_2_), 6.64 (s, 3-CH), 6.94 (d, 1H, J = 7.7 Hz, 29-CH), 7.08 (m, 14-CH), 7.09 (m, 15-CH), 7.18 (m, 11-CH), 7.21 (m, 28-CH), 7.39 (d, 1H, J = 7.9 Hz, 27-CH), 7.42 (s, 25-CH), 7.76 (s, 30-NH), 7.96 (s, 1-NH), 8.98 (t, 1H, J = 5.6 Hz, 17-NH); ^13^C-NMR (126 MHz, CDCl_3_): δ (ppm) 24.6 (32-CH_3_), 25.9 (19-CH_2_), 31.6 (9b-CH_2_), 32.2 (22,22′-(CH_2_)_2_), 39.3 (18-CH_2_), 42.0 (23-CH), 53.0 (3-CH), 54.1 (21,21′-(CH_2_)_2_), 56.3 (20-CH_2_), 59.1 (7-OCH_3_), 58.7 (9C-CH_2_OH), 61.5 (9a-OCH_2_), 68.1 (6-OCH_2_), 101.4 (4-C), 116.2 (d, J = 17.7, 11-CH), 117.4 (d, J = 17.3, 14-CH), 117.9 (27-CH), 118.1 (25-CH), 122.8 (29-CH), 123.1 (dd, J = 6.3, 3.6, 15-CH), 129.0 (28-CH), 137.6 (10-C), 138.3 (26-C), 146.5 (24-C), 146.6 (5-C), 150.0 (12- or 13-CF), 150.1 (12- or 13-CF), 152.2 (2-C), 153.3 (16-C), 164.5 (8-C), 168.6 (31-C); ^19^F-NMR (471 MHz, CDCl_3_): δ (ppm) -136.7 (m, 12- or 13-CF), -138.1 (m, 12- or 13-CF); MS: *m/z* (%) 658 (48), 345 (5), 302 (100), 259 (13), 231 (14), 160 (5), 113 (5); HRMS: Calcd. for C_33_H_47_F_2_N_5_O_7_Na [M + Na]^+^: 680.3271. Found: 680.2858.

## 4. Conclusions

Based on the increasing need for antiobesity drugs, two novel PET tracers for the MCHR1 have recently been developed, to investigate the role of the MCHR1 in terms of adiposity. The selective high-affinity MCHR1 antagonist SNAP-7941 **1** was used as a promising basis for the development of the PET tracers[^11^C]SNAP-7941 **1a** and [^18^F]FE@SNAP **4a** [1,15,16,17]. This paper focuses on the synthesis of the non-radioactive precursors and reference compounds of[^11^C]SNAP-7941 **1a** and [^18^F]FE@SNAP **4a**.

While the racemic receptor antagonist **1** [10,11,12,13,14] itself served as a reference compound for the preparation of[^11^C]SNAP-7941 **1a**, a new reference compound had to be synthesized for the novel fluoroethyled tracer [^18^F]FE@SNAP **4a** as already published by Philippe *et al.* [15,16]. The carboxylic acid derivative SNAP-acid **2** served as precursor for the ^11^C-methylation to afford the PET tracer[^11^C]SNAP-7941, while [^18^F]FE@SNAP was meant to be obtained in a first approach by ^18^F-fluorination of the newly prepared tosylate **3**.

The synthesis of these polyfunctional SNAP derivatives comprised many commonly used syntheses, including numerous methods attempting to cleave the methyl ester of **1** to prepare SNAP-acid **2**, which unfortunately were unsuccessful. Therefore, new approaches to obtain the desired derivatives accessible had to be established. A rationale for the failure of these demethylation methods could lay: (1) in the electron density which is distributed from the adjacent nitrogen to the carbonyl carbon through the conjugated double bond and thus, hinders the attack of nucleophiles (like OH^−^) and (2) in the circumstance that acidic conditions may affect the amide bonds. In summary, due to the failure of cleaving the methyl ester **1**, four different protecting groups (carboxyl esters) were chosen, leading to SNAP derivatives **29**–**32**. Finally, the precursor SNAP-acid **2** could be prepared through cleavage of the allyl protecting group of compound **32** [15,16]. Allyl-SNAP **32** was not only obtained in excellent yields compared with the other three protected derivatives, but was also used as starting material for two more SNAP derived compounds **35** and **36**.

As precursor for the tosylated compound **3**, HE@SNAP **35** was synthesized using three different methods, starting from either **32** or from the new derivatives **33** or **34**, respectively. Compound **35** was reacted to tosylate TOE@SNAP **3**, which could not be fluorinated in satisfying yields to furnish fluoroethyl ester FE@SNAP **4**. Additionally, in order to increase yields and feasibility of the fluorination step, a series of propylated compounds was prepared. Allyl ester **32** was reacted to the hydroxypropyl ester HP@SNAP **35**, followed by tosylation giving tosylpropyl ester TOP@SNAP **5**. Similarly to fluoroethyl ester FE@SNAP **4**, fluorination of **5** provided fluoropropyl ester FP@SNAP **6** only in low yields. The conversion was proved by mass spectrometry, but the isolation of **5** was hampered by decomposition during column chromatography.

Finally, fluoroethylation of the free SNAP-acid **2** (but not fluoropropylation) was achieved *via* Steglich esterification using DCC and DMAP. Thus, SNAP-acid **2** finally served as precursor for the radiosynthetically produced tracer[^11^C]SNAP-7941 **1a** as well as for the non-radioactive reference compound FE@SNAP **4** instead of tosylate **3**, which is used as precursor for the tracer [^18^F]FE@SNAP **4a** [15,16]. After radioactive labeling at the Medical University of Vienna [15,16], biodistribution and micro PET experiments will be the next step of this ongoing project, as recently shown by Philippe *et al.* [40].
